# Supplementary material for: Content-rich biological network constructed by mining PubMed abstracts
Source: BMC Bioinformatics. 2004 Oct 8;5:147. doi: 10.1186/1471-2105-5-147 (PMC528731; doi:10.1186/1471-2105-5-147)
Supplement: Additional File 2 — The original results of the above study (non-essential files are deleted to keep the file size under the limit set by BMC bioinformatics). [file 1471-2105-5-147-S2.bz2 › chilibotAdditionalFile2/dip10/481ID8702798E10571/html/IL8RB_CXCL5.html]

 


 **IL8RB** and **CXCL5** 
  
Found 16 abstracts in PubMed, retrieved 10.  
 

 What does Google say? 
 PDF only 
| .edu only 

---

**Interactive relationship** (e.g. stimulation, inhibition, etc)

**Neutral relationship**- CXCR2  [ **IL8RB** ]  binds IL 8 and other structurally related chemokines such as growth related oncogene GRO a, GRObeta, GROgamma, neutrophil activating peptide 2 NAP 2 and epithelial cell derived neutrophil activating peptide 78 ENA 78  [ **CXCL5** ] .  Ref: 12365735 Fish Shellfish Immunol, 2002

**Non-interactive relationship** (e.g. studied together, co-existance, homology, etc.)

- RESULTS Flow cytometry and radioligand binding data indicate that IL 8, GCP 2, and ENA 78  [ **CXCL5** ]  equivalently reduced CXCR 1 and CXCR 2  [ **IL8RB** ]  cell surface expression by 34% to 54%.  Ref: 10593335 Arch Surg, 1999
- Thus in COPD, in addition to CXCL8 and CXCR1,  **CXCL5**  and CXCR2  [ **IL8RB** ]  appear to play important roles in the airway neutrophilia characteristic of severe exacerbations.  Ref: 12857718 Am J Respir Crit Care MedAm J Respir Crit Care Med, 1999
- By contrast, the migratory response to the CXCR2  [ **IL8RB** ]  ligands, epithelial cell derived neutrophil activator ENA 78  [ **CXCL5** ]  and the growth related oncogene proteins, was markedly suppressed in PMN from septic patients p <.  Ref: 9973513 J Immunol, 1999
- By comparison with the NSC and S COPD groups, neutrophilia and gene expression for epithelial derived neutrophil attractant 78  **CXCL5** , interleukin 8 CXCL8, CXCR1 and CXCR2  [ **IL8RB** ]  were each up regulated in the E COPD group p&lt.  Ref: 12857718 Am J Respir Crit Care MedAm J Respir Crit Care Med, 1999
- CONCLUSION These findings suggest that, through expression of  **CXCL5** , eosinophils can recruit and activate CXC receptor 2 CXCR2  [ **IL8RB** ]  bearing cells such as neutrophils at sites of inflammation.  Ref: 12680872 Clin Exp Allergy, 2003
